# The True Story of *Yeti*, the “Abominable” Heterochromatic Gene of *Drosophila melanogaster*

**DOI:** 10.3389/fphys.2019.01093

**Published:** 2019-08-22

**Authors:** Yuri Prozzillo, Francesca Delle Monache, Diego Ferreri, Stefano Cuticone, Patrizio Dimitri, Giovanni Messina

**Affiliations:** ^1^Pasteur Institute of Italy, Fondazione Cenci Bolognetti, Rome, Italy; ^2^“Charles Darwin” Department of Biology and Biotechnology, Sapienza University of Rome, Rome, Italy

**Keywords:** *Drosophila melanogaster*, BCNT proteins, chromatin remodeling complex, YETI, epigenetic regulation

## Abstract

The *Drosophila Yeti* gene (CG40218) was originally identified by recessive lethal mutation and subsequently mapped to the deep pericentromeric heterochromatin of chromosome 2. Functional studies have shown that *Yeti* encodes a 241 amino acid protein called YETI belonging to the evolutionarily conserved family of Bucentaur (BCNT) proteins and exhibiting a widespread distribution in animals and plants. Later studies have demonstrated that YETI protein: (i) is able to bind both subunits of the microtubule-based motor kinesin-I; (ii) is required for proper chromosome organization in both mitosis and meiosis divisions; and more recently (iii) is a new subunit of dTip60 chromatin remodeling complex. To date, other functions of YETI counterparts in chicken (CENtromere Protein 29, CENP-29), mouse (Cranio Protein 27, CP27), zebrafish and human (CranioFacial Development Protein 1, CFDP1) have been reported in literature, but the fully understanding of the multifaceted molecular function of this protein family remains still unclear. In this review we comprehensively highlight recent work and provide a more extensive hypothesis suggesting a broader range of YETI protein functions in different cellular processes.

## Introduction

The eukaryotic genome is packaged into a highly condensed structure known as chromatin allowing cells to organize, compact and stabilize the genome into the nucleus. The first step of this process is achieved through nucleosome assembly which DNA into an 11 nm fiber that represents an approximate 6-fold level of compaction (Luger et al., 2012). Nucleosomes are octamers consisting of H2A/H2B and H3/H4 dimers which wrap around 147 bp of DNA. To gain access to DNA for replication, transcription and repair, nucleosomes must be shifted in different positions, removed or newly loaded to the DNA.

ATP-dependent protein complexes are the “molecular machines” having the task to specifically arrange the nucleosomal state. Thus far, all ATP-dependent chromatin-remodeling complexes identified contain a catalytic subunit which is part of the SWI2/SNF2 superfamily of ATPases and utilize a Swi2/Snf2-type ATPase domain consisting of an ATP-binding domain (DExx-domain), together with a helicase domain (HELICc-domain) (Clapier et al., 2017).

Four different classes (or families) of chromatin-remodeling complexes can be identified within chromatin remodeler ATPases superfamily: SWI/SNF, ISWI, CHD, and INO80 (Clapier et al., 2017). This classification is based on the presence of additional motifs outside the ATPase region. SWI/SNF  members  contain  a  bromodomain,  ISWI  members  contain  a  SANT  domain, while a

chromo- and a DNA-binding domain is found in CHD members. The INO80 class members do not contain any of these domains; instead, the ATPase domain is splitted into two segments (Lessard and Crabtree, 2010).

The process of chromatin remodeling generally refers to different changes in DNA–histone interaction within nucleosomes using the energy from ATP hydrolysis (Becker and Horz, 2002). They include repositioning of histone octamers in *cis* (along the same DNA template molecule) and in trans (from one DNA template molecule to another one), the loss of superhelical torsion of nucleosomal DNA, and the increase in accessibility to nucleosomal DNA for downstream processes as transcription. Interestingly, recent studies have shown that ATP-dependent chromatin remodeling may allow to change the histone composition of a nucleosome (Clapier and Cairns, 2009). Indeed, the yeast SWR1 complex, a member of the INO80 family, associates with Htz1, the homolog of mammalian histone variant H2A.Z. This complex can drive the ATP-dependent replacement of H2A–H2B with Htz1–H2B dimers (Krogan et al., 2003; Kobor et al., 2004; Mizuguchi et al., 2004; van Attikum and Gasser, 2005) by Swc1 ATPase subunit. *In vivo*, SWR1 catalyzes the incorporation of Htz1 into chromatin, which prevents the spreading of heterochromatin regions into regions of euchromatin (van Attikum and Gasser, 2005).

The human (SRCAP and P400/Tip60) and Drosophila (dTip60) orthologous chromatin remodeling complexes include proteins with high similarity to the subunits of two distinct chromatin-modifying complexes, the yeast NuA4 complex (Wang et al., 2018) harboring HAT (Histone Acetyl-transferase) activity, and the SWR1 ATP-dependent chromatin remodeling complex which catalyzes histones exchange (Table 1).

**TABLE 1:** SWR1 subfamily.

**Organism**	**Yeast**		**Fly**	**Human**		**Synonyms**	**Molecular function**
**Complex**	**NuA4**	**SWR1**	**Tip60**	**TRRAP/Tip60**	**SRCAP**		
ATPase	EAF1	SWR1	Domino	EP400	SRCAP	DOMO1, KIAA0309	Histone-tail binding/ATP-dependent helicase
Non-catalytic homolougous subunits		RVB1	Pontin	RUVBL1	RUVBL1	Pontin, ECP54, INO80H, NMP238, TIH1, Pontin52, TIP49, TIP49a	ATP-dependent helicase (unclear), scaffold
		RVB2	Reptin	RUVBL2	RUVBL2	Reptin, ECP51, INO80J, Reptin52, TIH2, TIP48, TIP49b	ATP-dependent helicase (unclear), scaffold
	ARP4	ARP4	BAP55	ACTL6A	ACTL6A	Actl6, BAF53A, INO80K	Phospho-H2A-variant-dependent DNA recruitment upon DNA damage
	ACT1	ARP6	Act87E	Actin	ACTR6	FLJ13433	Positive regulation of ATPase activity, actin related
	ESA1		Tip60	KAT5		cPLA2, PLIP, HTATIP1, ZC2HC5	HAT activity
	EAF3		MRG15	MORF4L1		HsT17725, MEAF3, MORFRG15	H3K36me2/3 binding
	EAF6		dEAF6	MEAF6		CENP-28, FLJ11730, NY-SAR-91	Unknown
	EAF7		dMRGBP	MRGBP		FLJ10914, MRG15BP	MRG15-binding, potential DNA-binding
	EPL1		E(pc)	EPC1			Protein-interaction within complex, regulation of HAT activity
	YNG2		dING3	ING3		Eaf4, FLJ20089, MEAF4, p47ING3	H3K4me3 binding
	TRA1		dTra1	TRRAP		PAF400, TR-AP	Adaptor, scaffold
	YAF9	YAF9	GAS41	YEATS4	YEATS4	NuBI-1	Transcriptional activation, nuclear matrix interaction
	SWC4	SWC4	dDMAP1	DMAP1	DMAP1	DNMAP1, DNMTAP1, EAF2, FLJ11543, KIAA1425, MEAF2	Histone-tail binding
		Bdf1	dBrd8	BRD8/TRCp120		p120, SMAP	Binding to acetylated histones, transcriptional coactivator
		VPS72	YL-1	VPS72	VPS72		H2A-variant binding
		H2AZ, H2B	H2A.V, H2B		H2AZ, H2B		Unknown
		VPS71			ZNHIT1	p18/Hamlet	Unknown
		SWC5	YETI		CFDP1	BCNT, CENP-29, CP27, p97	Unknown
Unique	EAF5, EAF1	SWC3, SWC7					Unknown

*Updated subunits composition of SWR1 chromatin remodeling complexes in yeast, fly and human according to FlyBase, HUGO gene nomenclature committee, yeastgenome databases and literature (Clapier and Cairns, 2009; Hargreaves and Crabtree, 2011; Rust et al., 2018).*

The dTip60 complex is made up by 14 core subunits (BAP55, dGAS41, dPontin, dReptin, Nipped-A, e(Pc), dYL1, dDMAP, Act87B, dMrg15, dMrgBP, dTRA1, dIng3, and dEaf6) and was found to be required for the replacement of acetylated phospho-H2A.V by unmodified H2A.V via Domino (Dom) ATPase (Kusch et al., 2004; March-Diaz and Reyes, 2009). In *Drosophila*, H2A.V is the only H2A variant and corresponds to mammalian H2A.X and H2A.Z (Baldi and Becker, 2013). Similarly to H2A.X, H2A.V is phosphorylated upon DNA double strand breaks to mark the DNA lesions, stimulate DNA repair machinery, and promote an easily accessible DNA conformation (Kusch et al., 2004).

The human P400/Tip60 complex highly resembles the dTip60 complex and contains the p400 protein, which also shows high sequence similarity to SWR1, DOMINO and SRCAP (Eissenberg et al., 2005; Table 1). Recent studies have demonstrated that several components of the P400/Tip60 complex are amplified or overexpressed in human neuroblastoma, glioblastoma, and colorectal cancer, while loss of function is mostly lethal, leading to cell growth arrest or cell death as well as genome instability (Yamada, 2012).

The YETI protein was found to be a new subunit of Drosophila Tip60 remodeling complex (Messina et al., 2014), arousing more and more curiosity because of a growing number of biological functions that it is supposed to perform.

Here, we comprehensively review emerging findings on YETI and its orthologous proteins in other species highlighting the broad-range involvement of these proteins in many biological processes.

## The Biological Role of Yeti Protein

Y*eti*, previously called CG40218, is an essential 1717-bp-long gene located in the heterochromatin of chromosome 2 of *Drosophila melanogaster* required for proper chromosome organization in both mitosis and meiosis (Hilliker, 1976; Corradini et al., 2003; Dimitri et al., 2003; Messina et al., 2014; Marsano et al., 2019). The evolutionary origin of *Yeti* gene is rather peculiar as such it has evolved from a euchromatic ancestor in drosophilids. The *Yeti* locus maps on euchromatin in two distantly related species, *D. pseudoobscura* and *D. virilis* indicating that over time (about 40 million of years, which is the estimated divergence time between *D. melanogaster* and *D. virilis*) it progressively moved closer to the heterochromatic pericentromeric regions (Moschetti et al., 2014; Caizzi et al., 2016). Interestingly, *Yeti* gene retained its original and robust organization in all analyzed species, with a short genomic region carrying a single short intron.

It is known that genes expressed at low levels harbor substantially shorter introns than those are expressed at high levels (Castillo-Davis et al., 2002). Therefore, despite of the genomic re-location, *Yeti* may have been under selective pressure maintaining a short size and ensuring high and efficient gene expression during early development (Moschetti et al., 2014).

**FIGURE 1:**
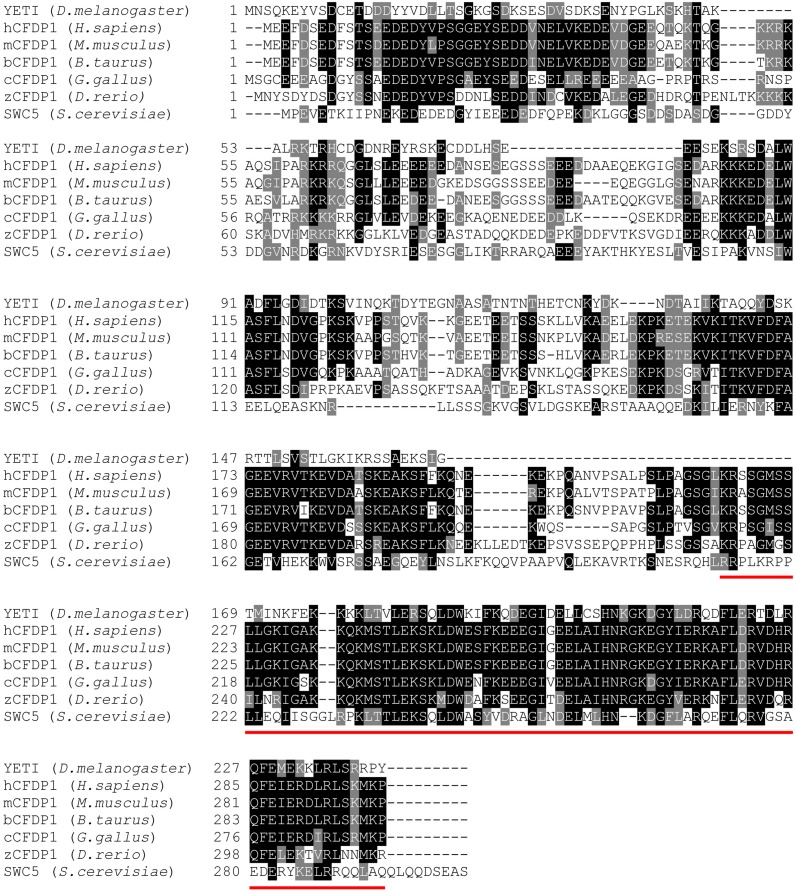
The YETI orthologous proteins. Blast alignments of YETI and its orthologous protein sequences from human (*H. sapiens*), mouse (*M. musculus*), bovine (*B. taurus*), chicken (*G. gallus*), Zebrafish (*D. rerio*) and yeast (*S. cerevisiae*). Note the high level of conservation (about 45% similarity) in the last 80 residues of the BCNT C-terminal domain (red line).

YETI is a 241-aa-long protein belonging to BCNT protein superfamily which is characterized by a yeast-to-human highly conserved 82-amino acid domain located at the C-terminus (BCNT-C) (Takahashi et al., 1998; Figure 1).

The YETI protein was originally identified as a both subunits kinesin-binding protein. In addition, Immunofluorescence experiments showed both a nuclear and cytoplasmic localization of V5 -tagged YETI in Drosophila S2 cells (Wisniewski et al., 2003). However, these experiments failed to clarify whether YETI is located within the nuclei or associated with the nuclear membrane.

**FIGURE 2:**
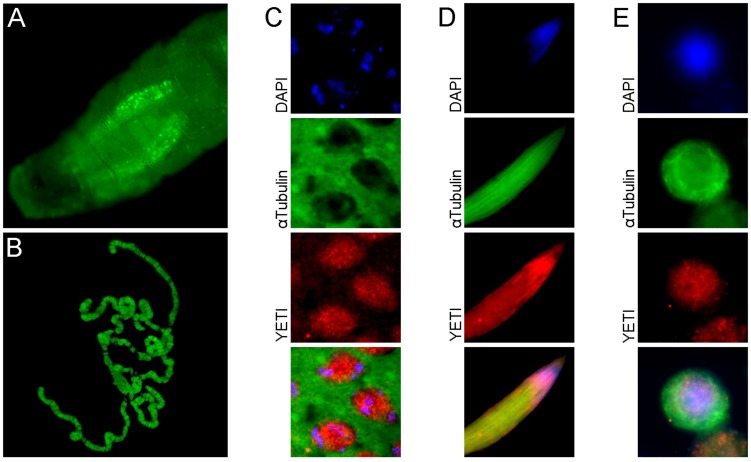
Subcellular YETI localization. **(A)** YETI::GFP fusion protein localizes in nuclei of salivary glands and **(B)** binds to polytene chromosomes. A nuclear immunolocalization of YETI protein is accordingly found in **(C)** spermatocytes, **(D)** elongated spermatids and **(E)** Drosophila S2 cells.

**FIGURE 3:**
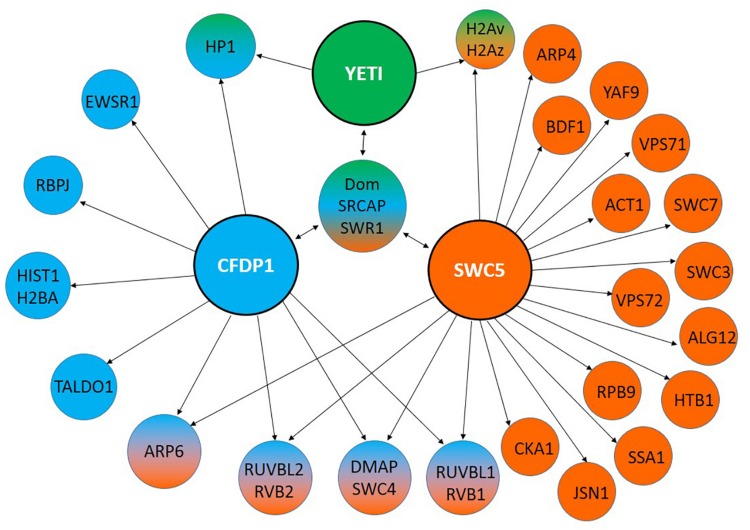
Interaction partners of YETI and its orthologs. YETI (*D. melanogaster*), hCFDP1 (*H. sapiens*) and SWR1 (*S. cerevisiae*) interacting proteins identified by using MIST (Molecular Interaction Search Tool). They refer only to physical interactions experimentally determined.

In the last decade, YETI protein functions have been investigated more in depth (Messina et al., 2014). In particular, YETI was found to be a chromatin-associated protein member of the dTip60 complex (Figure 2), whose depletion leads to larval stage lethality accompanied by profound perturbations of higher-order chromatin organization due to the failure in loading of histone H2A.V, nucleosomal histones, and chromatin marks. Moreover, the BCNT domain of YETI was found to be involved in chromatin binding through the interaction with the H2A histone variant (H2A.V), Domino-A (DOM-A) ATPase, and HP1a protein (Ryu et al., 2014; Figure 3).

Taken together, these results strongly suggest that YETI may be directly implicated in the exchange of the variant H2A.V onto nucleosomes impacting on the dynamic changes of chromatin, and fulfilling its role as subunit of Tip60 chromatin remodeling complex, similarly to the yeast homolog SWC5 (Morillo-Huesca et al., 2010).

Furthermore, in a genetic interaction map of cell cycle regulators, YETI (and also the DOMINO ATPase) has been defined as an additional interesting connection in the proximity of Anaphase-promoting complex/Cyclosome (APC/C) (Billmann et al., 2016), suggesting a specific role in the regulation of chromatin and transcription during mitosis (Morrison and Shen, 2009; Tanenbaum et al., 2015). Moreover, there are emerging evidence indicating that YETI is involved in synapse assembly and function (Pazos Obregon et al., 2015).

In conclusion, YETI appears to be a multifaceted chromatin protein playing a key role in different cellular pathways.

## The Molecular Function of Swc5, the Yeti Orthologous Protein in *S. cerevisiae*

Molecular insights of YETI orthologs come from other model organisms. For example, Swc5, the yeast ortholog protein, is a component of the SWR1 complex which catalyze the exchange of histone H2A with the H2A variant, Htz1 (Kobor et al., 2004; Mizuguchi et al., 2004). However, although Swc5 protein is not essential, *Swc5* null-mutants are defective in chromosome maintenance, and show decreased resistance to hydroxyurea (Saccharomyces Genome Database) and increased heat sensitivity (Dastidar et al., 2012).

*In vitro* studies report that the binding of SWR1 complex to Htz1 is not dependent on Swc5 subunit, but *Swc5* deleted mutants lack histone exchange function, indicating a key role of Swc5 during the histone replacement reaction (Wu et al., 2005). *In vivo* evidence supports this conclusion showing that the absence of Swc5 doesn’t affect complex assembly while it nearly dropped down the amount of Htz1 bound to chromatin, suggesting that Swc5 is required for the binding and destabilization of the H2A/H2B dimer (Morillo-Huesca et al., 2010). More in depth, SWR1 catalyzes H2A.Z *in vitro* replacement as one H2A.Z-H2B dimer at a time, generating heterotypic nucleosomes as intermediates and homotypic H2A.Z nucleosomes as final products. The SWR1 ATPase activity is specifically stimulated by H2A-containing nucleosomes, then free H2A.Z-H2B dimer leads to hyperstimulation of ATPase activity, eviction of nucleosomal H2A-H2B, and deposition of H2A.Z-H2B onto chromatin. In this way, SWR1 complex catalyzes nucleosomal conversion from H2A/H2A to Htz1/Htz1, with the heterotypic nucleosomes H2A/Htz1 as intermediates (Luk et al., 2010). Furthermore, a truncated Swc5 lacking the BCNT domain is not able to restore the reaction, indicating that BCNT domain is required for Htz1 deposition (Sun and Luk, 2017).

## Craniofacial Development Protein 1 (CFDP1): a “Developmental Face” of Yeti Orthologs in Mammals

A 97 kDa BCNT protein (p97Bcnt) was first identified in bovine brain and found to be widely distributed in animals and plants (Nobukuni et al., 1997; Takahashi et al., 1998; Iwashita and Naoki, 2011; Messina et al., 2015).

Later on, the murine ortholog was isolated and cloned from a mouse E11 (Embrional day 11) λgt11 library using an antibody against Cem1, a tooth cementum-related protein marker of a distinct expression pattern of mouse craniofacial development (Diekwisch et al., 1999). It maps on chromosome 8 in the E1 region and is 85 kb long, with a 7 exons (888 nucleotides) open reading frame transcribing a single mRNA and encoding a 295 a.a. protein of 27 kDa weight (mCFDP1). Due to its expression pattern and protein MW, the gene was originally named *craniofacial protein 27* (*cp27)* (Diekwisch et al., 1999).

Studies on the cp27 protein (later called mCFDP1) have shown specific interaction with NF-Y nuclear factor which bind CCAAT box (Luan et al., 2010; Ito et al., 2011), and with the TFII-I transcription factors involved in craniofacial development and osteo-differentiation (Makeyev and Bayarsaihan, 2011). Moreover, while RT-qPCR experiments revealed the presence of mCFDP1 in a pool of different genes related with transcription in osteo-differentiation (Bustos-Valenzuela et al., 2011), Northern blot analysis, combined with an *in situ* hybridization, have confirmed high level of its expression in developmental stage of mouse embryos, and in particular in neuroepithelium, cerebellum, heart, lung, liver, teeth, salivary glands, and periosteum of developing bones. These data have been also confirmed by immunohistochemical staining of developing tissues (Luan and Diekwisch, 2002).

Furthermore, cleft palate induction by persistent expression of PAX3 gene in neural crest correlates with cp27 downregulation (Wu et al., 2008).

The human BCNT ortholog maps to chromosome 16 in 16q22.2- q22.3, in proximity to several loci associated with inherited craniofacial disease genes (Diekwisch et al., 1999; Iwashita and Naoki, 2011). For this reason and for the expression pattern of the mouse ortholog it was called craniofacial development protein 1 gene (*Cfdp1*, OMIM number 608108). It is transcribed into two mRNAs differing by the alternative splicing-mediated inclusion or exclusion of exon encoding BCNT domain (Messina et al., 2017).

Independent experimental evidence suggests that the human CFDP1 (hCDFP1) protein is a subunit of the SNF2-related CBP activator protein (SRCAP) chromatin remodeling complex (Havugimana et al., 2012; Messina et al., 2017), which catalyzes the ATP-dependent replacement of canonical histone H2A with the H2A.Z variant (Monroy et al., 2001).

The first pioneer work aimed to address the mechanistic functions of hCFDP1 protein have been performed (Messina et al., 2016) by expressing human *Cfdp1* transgene in *D. melanogaster.* The results of this experiment have shown that *hCfdp1* expression lead to lethality and developmental and morphological defects, depending on the tissue-specific driver used. In particular, hCFDP1 binds to chromatin leading to abnormal polytene chromosome organization, decreased levels of H2A.V and H2A, brains and imaginal discs decreased size, developing of melanotic masses, prolonged larval stage and lethality. All these defects strongly resemble those produced by the lack of YETI (Messina et al., 2014). Thus, it is conceivable that hCFDP1 over-expression in *D. melanogaster* disrupts the physiological function of YETI resulting in a dominant-negative effect. Reciprocally, the *Drosophila* 3xFlag:YETI expressed in HeLa cells is able to go into the cell nucleus, bind to chromatin and produce a substantial decrease in the mitotic index with an important defects of cell cycle progression (Messina et al., 2016).

GST-pull down experiments have shown that both YETI and CFDP1 can undergo self-dimerization or hetero-dimerization. Thus, aberrant phenotypes could be due to the *in vivo* formation of YETI-CFDP1 non-functional hetero-dimers, together with a concomitant reduction of the amount of endogenous protein, YETI (drosophila cells) or CFDP1 (HeLa cells) (Messina et al., 2016; Messina et al., 2017).

Moreover, RNAi-mediated depletion of hCFDP1 in HeLa cells led to aberrant morphology and condensation defects of mitotic chromosomes impacting on their normal segregation during mitosis (Messina et al., 2017). These defects evoke those exhibited by *Yeti* null alleles in *Drosophila melanogaster*, suggesting a high-order chromatin organization maintenance function (Cenci et al., 2003; Messina et al., 2014).

Interestingly, the chromosome condensation defects observed in CFDP1 depleted cells resemble those found in cells lacking the MCPH1 gene encoding microcephalin, which is one of the causative genes of primary microcephaly (Yamashita et al., 2011; Messina et al., 2017).

Finally, in a human proteomic study, CFDP1 was found to interact with Ewing sarcoma related protein (EWSR1) whose mutations leads to Ewing’s sarcoma, a type of cancer that forms in bone or soft tissue (Bode-Lesniewska et al., 2019; Figure 3).

## Yeti Orthologs in Non-Mammalian Vertebrates

Among other vertebrates, data on YETI orthologs are only available from birds and fishes. In a recent study CENP-29, the chicken BCNT member, has been reported to be associated with kinetochores and therefore was suggested to play a role in chromosome segregation (Ohta et al., 2010).

In *Danio rerio*, the BCNT family gene, previously called *rltpr* (*RGD motif, leucine-rich repeats*, *tropomodulin domain* and *proline-rich containing*) and recently renamed *zCfdp1*, was found to be developmentally expressed in the eye, Central Nervous System and in branchial arches which lead to the craniofacial structures (Thisse and Thisse, 2004).

Recently, Celauro et al. (2017) have shown that zCFDP1 is rapidly produced after Maternal-To-Zygote transition and it is highly enriched in the head structures. Moreover, depletion of zCFDP1, induced by an ATG-blocking *morpholino*, produces drastic defects in craniofacial structures and bone mineralization.

Together, these results show that, not only that zCFDP1 is an essential protein required for proper embryonal development, but more importantly, they provide the first experimental evidence that it directly implicated in the morphogenesis of craniofacial territories in vertebrates. Moreover, based on the high level of identity (about 70%) (Figure 1), humans and zebrafish CFDP1 proteins, may be functionally conserved. It is then plausible that CFDP1, as a subunit of the SRCAP chromatin remodeling complex, participates to the epigenetic control of chromatin regions containing developmentally regulated genes, whose activation/silencing is crucial for proper differentiation of craniofacial structures and osteogenesis in zebrafish and likely in humans (Celauro et al., 2017).

## Emerging New Functions of Yeti and its Orthologs

As described above, the evolutionarily conserved Tip60 chromatin remodeling complex reveal multifaceted biological roles including transcriptional regulation, DNA repair, cell cycle progression, chromosome stability, stem cell maintenance and differentiation (Sapountzi et al., 2006; Tea and Luo, 2011).

As anticipated, depletion of YETI and CFDP1 in *D. melanogaster* and HeLa cells respectively affects mitotic index and led to chromosome segregation defects (Cenci et al., 2003; Messina et al., 2014, 2016). Moreover, the chicken YETI ortholog CENP-29 has been reported to be associated with kinetochores in chicken (Ohta et al., 2010). Finally, YETI protein has also been implicated in cell cycle control in both mitosis and meiosis (Billmann et al., 2016). According with these results, it has been reported that Myc, a well-known oncogenic player (Bellosta et al., 2005; Jonchere et al., 2017; Taylor et al., 2017), and the DOMINO ATPase interact each other and are required for normal asymmetric neuroblast (NB) division. Live-imaging analysis of *dom*-or *myc*-depleted NBs showed that spindle morphology is affected resulting in aberrant divisions (Rust et al., 2018). Similarly, Tip60 acetyl-transferase and Myc have been shown to co-regulate genes functionally involved in cell cycle and DNA replication (Zhao et al., 2018). Noteworthy, *D. melanogaster* mutants leading to symmetric division induce tumorigenesis (Albertson and Doe, 2003; Lee et al., 2006). Similarly, mutants lacking centrosomes and have severely impaired spindle asymmetry, over-proliferate and form tumors (Caous et al., 2015). Furthermore, mutations in genes encoding proteins directly or potentially implicated in chromatin biology are key players in human cancer and developmental disorders (Bickmore and van der Maarel, 2003; Fog et al., 2012; Harmacek et al., 2014).

Previous findings highlighted a role of YETI in spermatogenesis (Cenci et al., 2003). Accordingly, hCFDP1 has been found to physically interact with TALDO1 (Perl et al., 2006; Huttlin et al., 2015, 2017), an hallmark of human and murine spermatogenesis (Berkovits and Wolgemuth, 2013; Green et al., 2018), and HIST1H2BA (Figure 3), an intronless gene encoding a testis/sperm-specific member of the histone H2B family (Zalensky et al., 2002).

Taken together, the above-mentioned studies suggest that YETI and its orthologs, in addition to their canonical functions in chromatin remodeling, may play yet unexplored roles during both mitotic and meiotic cell division.

## Conclusion and Future Perspective

Most of the recent progresses in the study of the BCNT protein family originated from the functional analysis of a relatively small gene (*Yeti*) located in *D. melanogaster* constitutive heterochromatin, traditionally regarded as a transcriptionally inert or useless portion of the genome, albeit this prejudice has been debunked (Marsano et al., 2019). The fallout produced by those studies is relevant to basic research on epigenetics and to biomedical research too, as suggested by the experimental evidence showing that CFDP1 actively participates to craniofacial development in zebrafish. Craniofacial malformations are indeed developmental disorders of biomedical and clinical interest, because they represent the main cause of infant mortality and disability in humans. Therefore, it is reasonable to include the *Cfdp1* gene in the screening test for human syndromes showing alteration of craniofacial development.

## Author Contributions

PD and GM conceived the concept of the study and wrote the manuscript. YP and GM designed and formatted the figures and table. All authors drafted the literature search, edited and reviewed the manuscript.

## Conflict of Interest Statement

The authors declare that the research was conducted in the absence of any commercial or financial relationships that could be construed as a potential conflict of interest.
